# Carcass cutting yields and meat quality of market gilts managed with immunological suppression of ovarian function and estrus

**DOI:** 10.1093/tas/txae145

**Published:** 2024-10-05

**Authors:** Benjamin M Bohrer, Roy Edler, Lucina Galina Pantoja, Deborah Amodie, Martha A Mellencamp, Kimberly A Vonnahme

**Affiliations:** Department of Animal Sciences, The Ohio State University, Columbus, OH 43210, USA; Pipestone Applied Research, Pipestone, MN 56164, USA; Zoetis Inc., Parsippany, NJ 07054, USA; Zoetis Inc., Parsippany, NJ 07054, USA; Zoetis Inc., Parsippany, NJ 07054, USA; Zoetis Inc., Parsippany, NJ 07054, USA

**Keywords:** anti-GnRF, carcass cutting yield, estrus suppression, immunocastration, meat quality

## Abstract

The objective of this study was to analyze the effects of immunological suppression of ovarian function and estrus (Improvest®; Zoetis Inc.) on carcass cutting yields and meat quality. A total of 1,080 gilts were allocated by weight and assigned to pens of 27 pigs/pen. Pens were then randomly selected to be managed with or without immunological suppression of ovarian function and estrus (IMP vs. CON). Improvest was administered to each IMP gilt on days 7 and 67 of the study. Pigs were marketed on day 89 (*n* = 8 heaviest gilts per pen), 103 (*n* = 8 next heaviest gilts per pen), and 117 (remaining pigs/pen) of the study. The heaviest 47 to 50 carcasses for each treatment from each marketing group were selected for carcass cutting tests and evaluation of meat quality the day following slaughter (approximately 32-h postmortem). A smaller subset of 18 to 20 pork loins per treatment from each marketing group were selected for meat quality evaluation following 14 d of postmortem storage. Carcasses were fabricated according to institutional meat purchase specifications (IMPS) and meat quality analyses included pH and instrumental color at 32-h postmortem and purge loss, pH, instrumental color, intramuscular fat (IMF), cooking loss, and star probe following 14 d of postmortem storage. Live performance data were analyzed using the pen as the experimental unit, while carcass data were analyzed using the individual carcass as the experimental unit. During the post-second dose period (measured from days 70 to 85), IMP gilts consumed 10.8% more feed (*P* < 0.01) and grew 13.5% faster (*P* < 0.01) compared with CON gilts. Hot carcass weight (HCW), and the weights of many cuts, were significantly influenced by the interaction of Improvest management and marketing group. Therefore, the focus was directed toward primal weights and merchandized cut weights when expressed as a percentage of HCW. When expressed as a percentage of HCW, primal-cut yield and merchandized-cut yield calculations were not different (*P* = 0.33 and *P *= 0.65, respectively) between CON and IMP gilts. Meat quality traits did not differ (*P* > 0.05) between CON and IMP gilts, with the exception of instrumental a* at 32-h postmortem which was 0.32 units greater for CON gilts compared with IMP gilts. In summary, managing market gilts with immunological suppression of ovarian function and estrus improves the rate of weight gain through increased feed intake; however, carcass-cutting yields were not significantly changed.

## Teaser

This study evaluated the effect of immunological suppression of ovarian function and estrus (Improvest) on carcass cutting yields and meat quality. Overall, hot carcass weight and weights of cuts were significantly influenced by the interaction of Improvest management and the marketing group, illustrating the importance of marketing strategy when managing market gilts with Improvest. However, when expressed as a percentage of hot carcass weight, primal-cut yields and merchandized-cut yields were not different (*P*  = 0.33 and *P*  = 0.65, respectively) between CON and IMP gilts.

## Introduction

Improvest^®^ (marketed under the trade name of Improvac^®^, Innosure^®^, or Vivax^®^ in some parts of the world; Zoetis Inc., Parsippany, NJ, USA) is a gonadotropin-releasing factor (GnRF) analog-diphtheria toxoid conjugate formulated product approved for the temporary suppression of testicular function and reduction of boar taint in intact male pigs and for the temporary suppression of ovarian function and the suppression of estrus in intact female pigs. Managing market pigs with Improvest offers several secondary production advantages for both male pigs (Dunshea et al., 2001; Poulsen-Nautrup et al., 2018; Pérez-Ciria et al., 2022) and female pigs (Poulsen-Nautrup et al., 2020; Allison et al., 2021; Navasakuljinda et al., 2021), which ultimately results in reduced environmental burden of pig production attributed to improved feed efficiency (Poulsen-Nautrup et al., 2024).

Specific to managing market gilts with Improvest, several secondary production advantages can be obtained as a result of suppressing ovarian function, including a 10 to 15% improvement in weight gain during the final 3 to 7 wk of the finishing period (3% to 6% improvement in weight gain when calculated during the entire grow-finish period), which results in 3 to 5 kg heavier hot carcass weights (HCW) when compared to untreated gilts (Bohrer et al., 2014; Poulsen-Nautrup et al., 2020; Vasquez-Hidalgo et al., 2023). In addition, improvements in carcass weight consistency and group uniformity (i.e., less light weights) have been reported in mixed-sex populations of pigs (Bohrer et al., 2022). One particular gap in knowledge related to managing market gilts with Improvest is the impact on carcass-cutting yields, particularly that of trimmed, merchandized cuts. Poulsen-Nautrup et al. (2020), using a meta-analysis approach, reported that market gilts managed with Improvest were heavier (3.24 kg heavier HCW) and fatter (2.8 mm greater backfat thickness) than those not managed with Improvest; however, untrimmed primal weights were only different for the belly primal (raw mean differences of +0.02 kg for the ham primal, −0.06 kg for the shoulder primal, −0.06 kg for the loin primal, and +0.28 kg for the belly primal; trimmed primals were not reported in this study). This is a perplexing finding provided that the sum of these primals only accounts for +0.54 kg when the reported differences in HCW was +3.24 kg. This may be the product of the meta-analysis approach used in their study in addition to potential inconsistencies in cutting techniques employed among studies; nonetheless, additional research on carcass cutting yields for market gilts managed with Improvest is required. An additional component of managing market gilts with Improvest is the impact on meat quality attributes. It has been well documented that managing market gilts with Improvest does not significantly alter lean quality (i.e., color, pH, and water-holding capacity) but may improve fat quantity and quality (Bohrer et al., 2014; Poulsen-Nautrup et al., 2020; Vasquez-Hidalgo et al., 2024). Finally, evaluation of the interactive effects of managing market gilts with Improvest and marketing strategy has not been fully investigated nor described in previous studies.

Therefore, the primary objective of this study was to analyze the effects of immunological suppression of ovarian function and estrus (Improvest) on carcass cutting yields and meat quality, while conducting the experiment and analyzing the data in a manner which allowed for evaluation of the interactive effects of Improvest management and marketing group.

## Materials and Methods

The experiment was conducted at a commercial research grow-finish facility located in Minnesota, United States. Pigs were managed in accordance with the Pipestone Research Institutional Animal Care and Use Committee protocol ID 2021-9.

### Animals

At approximately 10 wk of age, 1,080 gilts (allocation weight of 32.7 ± 3.2 kg; Duroc sires [DNA 600; DNA Swine Genetics, Columbus, NE, USA] × white line sows [PIC Camborough; Genus PIC, Hendersonville, TN, USA]) were blocked into heavy-weight and light-weight blocks and assigned to 40 pens (7.2  × 2.6 m; 27 pigs/pen; initial stocking density of 0.69 m^2^/pig) in one room of a grow-finish barn. Pens were equipped with a 4-hole dry feeder (Crystal Spring Hog Equipment; Ste. Agathe, MB, Canada) and automatic drinkers (Hen-Way Manufacturing; Fairmont, MN, USA). The barn was ventilated with negative pressure and was maintained within thermo-neutral temperatures for grow-finish pigs. Pigs had *ad-libitum* access to feed and water throughout the duration of the grow-finish period. Diets were formulated to meet or exceed nutrient requirements according to NRC (2012) for market gilts (**Table 1**).

**Table 1.** Composition of the diets formulated for each dietary phase throughout the grow-finisher period

	Phase 1 (12 to 27 kg)	Phase 2 (27 to 36 kg)	Phase 3 (36 to 54 kg)	Phase 4 (54 to 72 kg)	Phase 5 (72 to 91 kg)	Phase 6 (91 to 104 kg)	Phase 7 (104 kg—market)
Ingredient, %							
Corn	56.74	57.11	62.52	67.53	70.82	79.90	82.67
Soybean meal	24.10	20.25	14.90	10.05	6.90	8.00	5.30
DDGS	15.00	20.00	20.00	20.00	20.00	10.00	10.00
Limestone	0.88	1.03	1.03	1.03	0.98	0.83	0.85
Monocalcium phosphate	0.80	—	—	—	—	—	—
Salt	0.60	0.50	0.50	0.50	0.50	0.50	0.50
Corn oil	0.58	—	—	—	—	—	—
Vitamin/trace mineral premix	0.15	0.15	0.15	0.15	0.15	0.15	0.15
l-Lysine	0.625	0.585	0.580	0.530	0.500	0.450	0.400
l-Threonine	0.250	0.200	0.180	0.155	0.125	0.140	0.105
dl-Methionine	0.165	0.110	0.075	0.025	—	0.005	—
l-Valine	0.085	0.035	0.030	—	—	—	—
l-Tryptophan	0.035	0.035	0.040	0.035	0.035	0.030	0.025
Calculated analysis^*^							
Crude protein, %	20.24	19.63	17.61	15.70	14.47	13.18	12.11
Crude fat, %	3.85	3.45	3.55	3.65	3.71	3.59	3.65
Crude fiber, %	3.75	3.9	3.69	3.51	3.39	2.93	2.82
Standardized ileal digestible amino acids, %							
Lysine	1.28	1.17	1.04	0.89	0.80	0.76	0.66
Isoleucine:lysine	55	57	57	57	58	57	59
Leucine:lysine	126	138	145	160	169	162	179
Methionine:lysine	36	34	33	30	30	29	30
Methionine + cysteine:lysine	59	59	59	58	59	58	64
Threonine:lysine	67	67	66	67	68	70	70
Tryptophan:lysine	16	16	16	16	16	16	15
Valine:lysine	70	69	69	70	71	70	74
Histidine:lysine	36	38	38	39	40	39	42
Net energy, kcal/kg	2,350	2,335	2,373	2,404	2,428	2,490	2,510
SID lysine:NE, g/Mcal	5.45	5.01	4.38	3.70	3.29	3.05	2.63

^*^Composition was calculated using EvaPig® software (French National Institute for Agriculture, Food and Environment Research [INRAE], METEX Animal Nutrition, and French Association of Zootechnie [AFZ]).

### Administration of Improvest

One-half of the pens of gilts (*n* = 20 pens; 540 gilts) were randomly assigned to be administered Improvest (IMP; a GnRF analog-diphtheria toxoid conjugate product; Zoetis Inc.) *via* injection by trained personnel according to manufacturer directions while the other half of the pens of gilts (*n* = 20 pens, 540 gilts) were not administered Improvest (CON). The first Improvest dose was administered on day 7 of the study (approximately 11 wk of age) and the second Improvest dose, which triggers the temporary suppression of ovarian function and estrus, was administered on day 67 of the study (approximately 19.5 wk of age).

### Collection of On-Farm Data

Pen weights and feed intake (calculated *via* feed disappearance) were collected on days 0, 27, 49, 70, and 85 of the study. Gain:Feed (G:F) was calculated as average daily gain (ADG) divided by average daily feed intake (ADFI). Mortality and removals as well as full-value, lights, and culls were determined by study personnel and recorded on a per-animal basis.

### Marketing and Slaughter Procedures

Pigs were marketed in 3 marketing groups with the 8 heaviest gilts in each pen marketed on day 89 of the study (22 d post-second Improvest dose), the 8 next heaviest gilts in each pen marketed on day 103 of the study (36 d post-second Improvest dose), and the remaining pigs marketed on day 117 of the study (50 d post-second Improvest dose). Theoretically (not accounting for mortality and removals), the average time post-second Improvest dose for IMP gilts was 37.6 d. Pigs selected for marketing were individually weighed and the remaining pen weight was collected following their removal.

Pigs were slaughtered under commercial conditions and individual pig identification was maintained throughout the slaughter process. Head-removed HCWs were recorded by processing plant personnel and carcass dressing percentage was calculated as HCW divided by weight at marketing. Backfat thickness was collected at the last rib location using a ruler.

### Carcass Cutting Tests

For each marketing group, a target of 50 carcasses per treatment (actual values were 47 to 50 carcasses per treatment) were collected for a carcass cutting test at approximately 32-h postmortem (pigs were harvested at 0600 hours and tests were conducted the following afternoon). These carcasses were selected to include the heaviest carcasses from each treatment. Primal-cut (IMPS #401A ham, IMPS #405 picnic, IMPS #406 butt, IMPS #408 belly, IMPS #410 loin, and IMPS #416 spareribs) and merchandized-cut (IMPS #405A picnic, IMPS #406A butt, IMPS #409 belly, IMPS #414 Canadian back loin, and IMPS #416A spareribs) specifications followed the packing plant matrix where the study was conducted and coincided with the institutional meat purchase specifications (IMPS; NAMP, 2006; IMPS, 2014). Two cutting yield equations were used to capture untrimmed primal-cut yields and merchandized-cut yields:

Primal-cut yield = [(2 × (401A ham weight + 405 picnic weight + 406 butt weight + 408 belly weight + 410 loin weight + 416 spareribs weight)) / hot carcass weight] × 100

Merchandized-cut yield = [(2 × (401A ham weight + 405A picnic weight + 406A butt weight + 408 belly weight + 414 loin weight + 416A spareribs weight)) / hot carcass weight] × 100.

### Meat Quality Analysis

pH (Sentron SI600; Norlab, Helsinki, Finland) and instrumental color (Minolta CR-400 with illuminant D_65_, an 8-mm aperture, and 0° viewing angle; Konica Minolta Sensing Americas, Inc, Ramsey, NJ, USA) was collected on the boneless loins for the carcasses used for the cutting tests immediately following the cutting tests (approximately 32-h postmortem). From this population, 18 to 20 boneless loins were randomly selected for analysis of aged meat quality. For aged meat quality tests, loins were vacuum-packaged and aged for an additional 14 d postmortem. Purge loss was measured when loins were removed from their vacuum packages. pH (Hanna HI9025; Hanna Instruments, Woonsocket, RI, USA), instrumental color (Minolta CR-410 with illuminant D_65_, a 50-mm aperture, and 2° viewing angle; Konica Minolta Sensing Americas, Inc), intramuscular fat (IMF) *via* rapid NMR fat analyzer (CEM Corporation, Charlotte, NC, USA), and Instron Star-probe (Instron; Grove City, PA, USA) was subsequently measured on the aged pork loins following 14 d of postmortem storage.

### Statistical Analysis

Live performance data (ADFI, ADG, and G:F) were analyzed using the pen as the experimental unit. A linear mixed model approach for repeated measures with PROC MIXED of SAS (v. 9.4, SAS Inst. Inc.; Cary, NC, USA) was utilized with fixed effects of Improvest management, day, and their interaction and the random effect of block. Mortality and removals, full values, lights, and culls were recorded on a per-animal basis. These data were defined as binary variables (1 = yes, 0 = no) and analyzed by a linear mixed model approach using PROC GLIMMIX of SAS. Carcass data (carcass cutting tests and meat quality data) were analyzed using the individual carcass as the experimental unit. A linear mixed model approach with PROC MIXED of SAS was utilized with fixed effects of Improvest management, marketing group, and their interaction and the random effect of pen. Least squares means were separated using the PDIFF option in SAS. Differences for fixed effects were considered significant at *P* ≤ 0.05.

## Results and Discussion

### Live Performance

There were no differences (*P* > 0.05) for live performance parameters (live weight, ADFI, ADG, or G:F) prior to day 70 of the study, which was expected as the second Improvest dose was administered on day 67 of the study and all responses attributed to the study treatment would be seen following this point of the study (**Table 2**). The final collection of live weights and feed intake for this study were on day 85 (18 d post-second Improvest dose; 4 d before marketing group #1). On this day, IMP gilts were 1.8 kg heavier than CON gilts, which coincided with 10.8% greater ADFI (*P* < 0.01) and 13.5% greater ADG (*P* < 0.01) for IMP gilts when compared with CON gilts from days 70 to 85. These findings are supported by previous research studies evaluating the live performance of gilts managed with immunological suppression of ovarian function and estrus, including Bohrer et al. (2024), which reported Improvest gilts were 4.1 kg heavier, had 11.8% greater ADFI, and had 15.3% greater ADG during the first 3 wk following the second Improvest dose. One component that is missing from the current study is live performance during the marketing period (i.e., the time period following the first 18 d post-second Improvest dose; day 85 to the final marketing group on day 117). It can be assumed based on data from other studies that improvements in ADG would continue throughout the study yet feed consumption would begin to outpace the growth rate resulting in the feed efficiency of IMP gilts likely being lower than that of CON gilts. In support of this assumption, Bohrer et al. (2024) reported Improvest gilts consumed 19.5% more feed, grew 5.7% faster, and were less efficient (11.6% lower G:F) compared with CON gilts during the second 3-wk interval of the post-Improvest period (week-3 to week-6 during which the heaviest pigs from each pen were being marketed).

**Table 2.** Effect of immunological suppression of ovarian function and estrus (Improvest) of market gilts on live performance characteristics (whole population analysis)

	Improvest management^*^		
	CON	IMP	SEM
Number of pens (pigs)^†^, no.	20 (540)	20 (540)	
Live weight by day^‡^, kg			
day 0	32.69	32.71	2.56
day 27	57.51	57.65	2.57
day 49	78.11	78.13	2.57
day 70	99.37	99.63	2.59
day 85	110.97^b^	112.80^a^	2.59
Average daily feed intake^‖^, kg/d			
days 0 to 27	1.83	1.82	0.06
days 27 to 49	2.12	2.14	0.07
days 49 to 70	2.53	2.55	0.07
days 70 to 85	2.39^b^	2.68^a^	0.07
days 0 to 85	2.22^b^	2.36^a^	0.02
Average daily gain^$^, kg/d			
days 0 to 27	0.91	0.92	0.01
days 27 to 49	0.89	0.91	0.02
days 49 to 70	1.00	1.01	0.02
days 70 to 85	0.74^b^	0.84^a^	0.03
days 0 to 85	0.88^b^	0.93^a^	0.01
Gain:Feed^¶^			
days 0 to 27	0.501	0.506	0.012
days 27 to 49	0.417	0.428	0.013
days 49 to 70	0.397	0.395	0.011
days 70 to 85	0.309	0.313	0.013
days 0 to 85	0.398	0.394	0.011
Final outcomes^**^			
Full value, %	95.86	97.17	0.26
Dead/removed, %	2.83	1.70	0.24
Lights, %	0.47	0.18	0.44
Culls, %	0.71	0.90	0.74

^ab^LSMeans ± SEM within a time period with different superscripts differ (*P* ≤ 0.05).

^*^Improvest is a gonadotropin-releasing factor (GnRF) analog-diphtheria toxoid conjugate product approved for temporary suppression of ovarian function and estrus in market gilts (Zoetis Inc.); Improvest (IMP) gilts received the first Improvest injection on day 7 of the study (approximately 11 wk of age) and the second Improvest injection on day 67 of the study (approximately 19.5 wk of age); Control (CON) gilts did not receive Improvest injections.

^†^This was the number of gilts that started the study (i.e., does not account for removals or missing datapoints).

^‡^Repeated measures analysis *P*-values: Treatment: *P* = 0.22; Day *P* < 0.001; Treatment × Day: *P* = 0.001.

^‖^Repeated measures analysis *P*-values: Treatment: *P* < 0.001; Day *P* < 0.001; Treatment × Day: *P* < 0.001.

^$^Repeated measures analysis *P*-values: Treatment: *P* = 0.005; Day *P* < 0.001; Treatment × Day: *P *= 0.10.

^¶^Repeated measures analysis *P*-values: Treatment: *P* = 0.31; Day *P* < 0.001; Treatment × Day: *P* = 0.62.

^**^Main effect treatment *P* values for final outcomes: *P* ≥ 0.24.

SEM, standard error of the mean.

There were no statistical differences (*P* > 0.05) between CON and IMP gilts for the marketing of full-value pigs, deads/removals, lights, or culls. In specific regard to the percentage of full-value pigs, limited available data may suggest improved marketing uniformity of gilts managed with immunological suppression of ovarian function and estrus when compared with untreated gilts. For instance, a study conducted by Bohrer et al. (2022) reported that the uniformity of carcass weight was improved when gilts were managed with immunological suppression of ovarian function and estrus when compared with untreated gilts (the percentage of gilt carcasses weighing less than 90 kg were 6.7% for Improvest gilts and 18.0% for untreated gilts). This particular measurement requires greater consideration in future research studies, and it would be most appropriate to measure and quantify this parameter in large populations of pigs raised in commercial settings.

### Carcass Characteristics

Carcass characteristics were collected on the entire population of pigs (**Table 3**) while the cutting and meat quality tests were collected on a sub-population of the pigs. For the entire population of pigs, there were significant interactions (*P* < 0.05) between the Improvest management and marketing group for market weight, HCW, and backfat thickness while there was not a significant interaction (*P* = 0.70) between Improvest management and marketing group for carcass dressing percentage. The interactions for market weight and HCW can be described as differing magnitudes of change between CON and IMP gilts at each of the marketing groups. For instance, the IMP gilts marketed on day 89 were 0.90 kg heavier for their market weight and 0.17 kg heavier for their HCW compared to the CON gilts marketed on day 89, the IMP gilts marketed on day 103 were 4.48 kg heavier for their market weight and 2.61 kg heavier for their HCW compared to the CON gilts marketed on day 103, and the IMP gilts marketed on day 117 were 6.72 kg heavier for their market weight and 4.10 kg heavier for their HCW compared to the CON gilts marketed on day 117. This demonstrates a lack of a treatment response for weights in the first marketing group and a much stronger treatment response for weights in the second and third marketing groups. This was observed in another study (Vasquez-Hidalgo et al., 2023) where the response levels for HCW of Improvest gilts vs. untreated gilts were as follows: marketing group #1: + 0.77 kg; marketing group #2: + 2.53; marketing group #3: + 3.73 kg; marketing group #4: + 5.77 kg. The interaction for backfat thickness can also be described as differing magnitudes of change between CON and IMP gilts at each of the marketing groups, and closely aligns with the observations for weights. The IMP gilts marketed on day 89 were 0.7 mm fatter compared to the CON gilts marketed on day 89, the IMP gilts marketed on day 103 were 2.0 mm fatter compared to the CON gilts marketed on day 103, and the IMP gilts marketed on day 117 were 2.6 mm fatter compared to the CON gilts marketed on day 117.

**Table 3.** Effect of immunological suppression of ovarian function and estrus (Improvest) of market gilts marketed over 3 marketing groups [day 89 (22 d post-second dose), day 103 (37 d post-second dose), and day 117 of the study (day 50 post-second dose)] on market weight and carcass characteristics (whole population analysis)

	Interaction of Improvest Management and Marketing Group^*^							The main effect of improvest management					
	CON			IMP							*P*-value		
	day 89	day 103	day 117	day 89	day 103	day 117	SEM	CON	IMP	SEM	Improvest management	Marketing group	Interaction
Number of pigs, no.	160	160	189	160	160	195		509	515				
Market weight, kg	123.90^d^	127.46^c^	126.26^bc^	124.80^cd^	131.94^a^	132.98^a^	2.88	125.87^y^	129.91^x^	2.83	<0.001	<0.001	<0.001
Hot carcass weight, kg	91.69^d^	93.66^c^	93.78^c^	91.86^d^	96.27^b^	97.88^a^	2.19	93.05^y^	95.33^x^	2.15	0.001	<0.001	<0.001
Carcass dressing percentage	74.0^a^	73.4^c^	73.9^ab^	73.7^b^	73.0^d^	73.7^b^	0.1	73.8^x^	73.5^y^	0.1	<0.001	<0.001	0.70
Backfat thickness^†^, mm	26.2^bc^	23.6^d^	25.2^cd^	26.9^ab^	25.6^c^	27.8^a^	0.1	25.0^y^	26.8^x^	0.7	<0.001	<0.001	0.02

^abcd^LSMeans ± SEM within a row of interaction means with different superscripts differ; *P* ≤ 0.05.

^xy^LSMeans ± SEM within a row of the main effect means with different superscripts differ; *P* ≤ 0.05.

^*^Improvest is a gonadotropin-releasing factor (GnRF) analog-diphtheria toxoid conjugate product approved for temporary suppression of ovarian function and estrus in market gilts (Zoetis Inc.); Improvest (IMP) gilts received the first Improvest injection on day 7 of the study (approximately 11 wk of age) and the second Improvest injection on day 67 of the study (approximately 19.5 wk of age); Control (CON) gilts did not receive Improvest injections; An equal number of CON and IMP gilts were marketed on d 89 (approximately 30% of the population), day 103 (approximately 30% of the population), and d 117 (approximately 40% of the population) of the study.

^†^Backfat thickness was collected at the last rib location using a ruler.

SEM, standard error of the mean.

There were significant main effects for market weight, HCW, carcass dressing percentage, and backfat thickness attributed to Improvest management. To summarize, IMP gilts were heavier (4.04 kg heavier market weight and 2.28 kg heavier HCW; *P* < 0.01), had greater carcass dressing percentage (0.3 percentage units greater; *P* < 0.01), and were fatter (1.8 mm greater backfat thickness; *P *< 0.01) compared with CON gilts. These response levels align with previous studies that have reported heavier weights and greater backfat thickness for market gilts managed with Improvest compared with untreated gilts (Poulsen-Nautrup et al., 2020; Vasquez-Hidalgo et al., 2023; Bohrer et al., 2024).

There were significant main effects for market weight, HCW, carcass dressing percentage, and backfat thickness attributed to the marketing group. To summarize, gilts in marketing group #1 were lighter than gilts in marketing group #2 and marketing group #3, while gilts in marketing group #2 had lower carcass dressing percentage and were trimmer compared with gilts in marketing group #1 and marketing group #3. Inconsistencies in weights and carcass characteristics like backfat thickness have been reported previously (Arkfeld et al., 2016; Rickard et al., 2017). A goal for production systems utilizing multiple marketing groups should be to ensure that market weights (and inherently HCW) are reaching or exceeding targets and that weights for each marketing group are fairly uniform (Zhou and Bohrer, 2019); thus, the pigs in this study likely missed their target for the first marketing group.

### Carcass Cutting Yields

HCW for the 291 carcasses used in the cutting tests and untrimmed primal weights were presented in **Table 4**. There was a significant interaction (*P* < 0.01) between Improvest management and the marketing group for HCW. This interaction can be described as differing magnitudes of change between CON and IMP gilts at each of the marketing groups. For instance, the IMP gilts marketed on day 89 were 1.28 kg lighter for their HCW compared to the CON gilts marketed on day 89, the IMP gilts marketed on day 103 were 1.74 kg heavier for their HCW compared to the CON gilts marketed on day 103, and the IMP gilts marketed on day 117 were 4.86 kg heavier for their HCW compared to the CON gilts marketed on day 117. This demonstrates a challenge associated with the selection criteria for the carcasses used (selection for the heaviest from each treatment) for the cutting tests in this study but nonetheless does provide a useful model for industrial purposes. The numerical difference for HCW observed between IMP gilts and CON gilts of 1.77 kg was not significant (*P* = 0.09). There was a significant difference for HCW observed among the marketing groups with marketing group #1 being the lightest (94.6 kg), marketing group #2 being intermediate (97.5 kg), and marketing group #3 being the heaviest (100.9 kg).

**Table 4.** Effect of immunological suppression of ovarian function and estrus (Improvest) of market gilts marketed over 3 marketing groups (day 89 [22 d post-second dose], day 103 [37 d post-second dose], and day 117 of the study [day 50 post-second dose]) on untrimmed (i.e., natural fall) primal weights

	Interaction of Improvest Management and Marketing Group^*^							The main effect of improvest management					
	CON			IMP							*P*-value		
	day 89	day 103	day 117	day 89	day 103	day 117	SEM	CON	IMP	SEM	Improvest management	Marketing group	Interaction
Number of carcasses, no.	47	49	50	49	49	47		146	145				
Hot carcass weight, kg	95.21^cd^	96.59^bc^	98.45^b^	93.93^d^	98.33^b^	103.31^a^	0.95	96.75	98.52	0.73	0.09	<0.001	<0.001
Cut (IMPS #)^†^													
Ham (401A), kg	11.12^bc^	11.19^bc^	11.24^bc^	10.99^c^	11.45^ab^	11.62^a^	0.12	11.18	11.35	0.08	0.15	0.002	0.05
Picnic (405), kg	4.83^c^	4.95^bc^	5.50^a^	5.03^b^	4.98^bc^	5.68^a^	0.07	5.09^y^	5.23^x^	0.04	0.02	<0.001	0.41
Butt (406), kg	4.12^c^	4.56^b^	4.52^b^	4.17^c^	4.62^b^	4.86^a^	0.08	4.40^y^	4.55^x^	0.05	0.05	<0.001	0.06
Belly (408), kg	8.88^c^	9.20^ab^	8.93^bc^	8.66^c^	9.50^a^	9.42^a^	0.13	9.01	9.19	0.10	0.18	<0.001	0.002
Loin (410), kg	12.19^bc^	12.25^b^	12.39^b^	11.84^c^	12.46^b^	13.09^a^	0.15	12.28	12.46	0.10	0.20	<0.001	<0.001
Spareribs (416), kg	2.72^c^	2.65^c^	2.84^b^	2.61^c^	2.65^c^	3.12^a^	0.04	2.74	2.79	0.03	0.16	<0.001	<0.001
Sum of untrimmed primal weights per carcass^‡^, kg	87.70^cd^	89.56^bc^	90.82^b^	86.50^d^	91.23^b^	95.62^a^	0.89	89.36	91.12	0.68	0.07	<0.001	<0.001

^abcd^LSMeans ± SEM within a row of interaction means with different superscripts differ; *P* ≤ 0.05.

^xy^LSMeans ± SEM within a row of the main effect means with different superscripts differ; *P* ≤ 0.05.

^*^Improvest is a gonadotropin-releasing factor (GnRF) analog-diphtheria toxoid conjugate product approved for temporary suppression of ovarian function and estrus in market gilts (Zoetis Inc.); Improvest (IMP) gilts received the first Improvest injection on day 7 of the study (approximately 11 wk of age) and the second Improvest injection on day 67 of the study (approximately 19.5 wk of age); Control (CON) gilts did not receive Improvest injections; An equal number of CON and IMP gilts were marketed on day 89 (approximately 30% of the population), d 103 (approximately 30% of the population), and day 117 (approximately 40% of the population) of the study.

^†^Institutional meat purchase specifications (IMPS, 2014).

^‡^Sum of untrimmed primal weights per carcass = [2 × (401A ham weight + 405 picnic weight + 406 butt weight + 408 belly weight + 410 loin weight + 416 spareribs weight)].

SEM, standard error of the mean.

In terms of weights for untrimmed primals, there was a significant interaction (*P* ≤ 0.05) between the Improvest management and marketing group for the untrimmed ham, untrimmed belly, untrimmed loin, untrimmed spareribs, and for the sum of the untrimmed primal weights while there was not a significant interaction (*P* > 0.05) for the untrimmed picnic and untrimmed butt weights. The significant interactions were largely attributed to the aforementioned differences observed for HCW. While all untrimmed cuts were numerically heavier for IMP gilts compared with CON gilts, which culminated in the sum of untrimmed primal weights being 1.76 kg heavier (*P* = 0.07), only the untrimmed picnic and untrimmed butt cuts were significantly different (*P* < 0.05) between IMP and CON gilts. There was a significant difference (*P* < 0.01) for all untrimmed primal-cut weights observed among the marketing groups.

Untrimmed primal yields expressed as a percentage of HCW were presented in **Table 5**. There was a significant interaction (*P* ≤ 0.05) between Improvest management and the marketing group for the untrimmed picnic and untrimmed spareribs when expressed as a percentage of HCW while there was not a significant interaction (*P* > 0.05) for the untrimmed ham, untrimmed butt, untrimmed belly, untrimmed loin, or the sum of the untrimmed primal cuts when expressed as a percentage of HCW. There were no effects (*P* > 0.05) for Improvest management on the untrimmed primal cuts when expressed as a percentage of HCW. There was a significant difference (*P* < 0.01) among the marketing groups for all untrimmed primal cut weights when expressed as a percentage of HCW with the lone exception for the untrimmed loin.

**Table 5.** Effect of immunological suppression of ovarian function and estrus (Improvest) of market gilts marketed over 3 marketing groups (day 89 [22 d post-second dose], day 103 [37 d post-second dose], and day 117 of the study [day 50 post-second dose]) on untrimmed (i.e., natural fall) primal weights expressed as a percentage of hot carcass weight (HCW)

	Interaction of Improvest Management and Marketing Group^*^							The main effect of improvest management					
	CON			IMP							*P-*value		
	day 89	day 103	day 117	day 89	day 103	day 117	SEM	CON	IMP	SEM	Improvest management	Marketing group	Interaction
Number of carcasses, no.	47	49	50	49	49	47		146	145				
Cut (IMPS #)^†^													
Whole ham (401A), % of HCW	23.38^a^	23.18^ab^	22.85^bc^	23.41^a^	23.27^ab^	22.52^c^	0.16	23.14	23.07	0.09	0.60	<0.001	0.37
Picnic (405), % of HCW	10.13^c^	10.24^c^	11.18^a^	10.66^b^	10.09^c^	11.01^a^	0.12	10.52	10.59	0.07	0.47	<0.001	0.003
Butt (406), % of HCW	8.64^c^	9.43^a^	9.16^ab^	8.86^bc^	9.38^a^	9.40^a^	0.12	9.08	9.21	0.07	0.16	<0.001	0.38
Belly (408), % of HCW	18.65^bc^	19.02^ab^	18.13^d^	18.42^cd^	19.34^a^	18.21^d^	0.15	18.60	18.66	0.10	0.67	<0.001	0.17
Loin (410), % of HCW	25.59	25.35	25.15	25.21	25.34	25.37	0.21	25.36	25.31	0.12	0.73	0.79	0.32
Spareribs (416), % of HCW	5.71^bc^	5.49^d^	5.78^b^	5.56^cd^	5.38^d^	6.03^a^	0.08	5.66	5.66	0.04	0.97	<0.001	0.01
Primal-cut yield^‡^, % of HCW	92.09^c^	92.72^ab^	92.26^bc^	92.12^c^	92.80^a^	92.56^abc^	0.17	92.36	92.49	0.10	0.33	0.001	0.71

^abcd^LSMeans ± SEM within a row of interaction means with different superscripts differ; *P* ≤ 0.05.

^xy^LSMeans ± SEM within a row of the main effect means with different superscripts differ; *P* ≤ 0.05.

^*^Improvest is a gonadotropin-releasing factor (GnRF) analog-diphtheria toxoid conjugate product approved for temporary suppression of ovarian function and estrus in market gilts (Zoetis Inc.); Improvest (IMP) gilts received the first Improvest injection on day 7 of the study (approximately 11 wk of age) and the second Improvest injection on day 67 of the study (approximately 19.5 wk of age); Control (CON) gilts did not receive Improvest injections; An equal number of CON and IMP gilts were marketed on day 89 (approximately 30% of the population), day 103 (approximately 30% of the population), and day 117 (approximately 40% of the population) of the study.

^†^Institutional meat purchase specifications (IMPS, 2014).

^‡^Primal-cut yield = [(2 × (401A ham weight + 405 picnic weight + 406 butt weight + 408 belly weight + 410 loin weight + 416 spareribs weight))/hot carcass weight] × 100.

SEM, standard error of the mean.

Trimmed primal weights were presented in **Table 6**. There was a significant interaction (*P* ≤ 0.05) between Improvest management and the marketing group for the trimmed and squared belly, the Canadian back loin, and the trimmed spareribs while there was not a significant interaction (*P* > 0.05) for the trimmed picnic, trimmed butt, or the sum of the trimmed cuts. While all trimmed cuts were numerically heavier for IMP gilts compared with CON gilts, which culminated in the sum of trimmed primal weights being 1.07 kg heavier (*P* = 0.08), only the trimmed picnic cut was significantly different (*P* = 0.01) between IMP and CON gilts. There was a significant difference (*P* < 0.01) for all trimmed primal cut weights observed among the marketing groups with the lone exception for the trimmed and squared belly.

**Table 6.** Effect of immunological suppression of ovarian function and estrus (Improvest) of market gilts marketed over 3 marketing groups (day 89 [22 d post-second dose], day 103 [37 d post-second dose], and day 117 of the study [day 50 post-second dose]) on trimmed (i.e., finished good/merchandized) primal weights

	Interaction of Improvest Management and Marketing Group^*^							The main effect of improvest management		*P*-value			
	CON			IMP									
	day 89	day 103	day 117	day 89	day 103	day 117	SEM	CON	IMP	SEM	Improvest management	Marketing group	Interaction
Number of carcasses, no.	47	49	50	49	49	47		146	145				
Cut (IMPS #)^†^													
Picnic, trimmed (405A), kg	3.90^c^	4.06^b^	4.53^a^	4.08^b^	4.13^b^	4.63^a^	0.06	4.16^y^	4.28^x^	0.03	0.011	<0.001	0.60
Butt, trimmed (406A), kg	3.56^c^	3.95^b^	3.97^b^	3.60^c^	4.00^b^	4.22^a^	0.07	3.83	3.94	0.05	0.102	<0.001	0.13
Belly, trimmed and squared (409), kg	5.90^ab^	5.84^b^	5.68^b^	5.74^b^	6.18^a^	6.00^ab^	0.12	5.81	5.98	0.09	0.17	0.13	0.023
Loin, Canadian back (414), kg	3.74^bc^	3.73^bc^	3.76^ab^	3.61^c^	3.72^bc^	3.90^a^	0.05	3.74	3.74	0.03	0.94	0.004	0.026
Spareribs, trimmed (416A), kg	2.40^c^	2.37^c^	2.51^b^	2.35^c^	2.33^c^	2.66^a^	0.04	2.43	2.45	0.02	0.51	<0.001	0.003
Sum of merchandized weights per carcass^‡^, kg	48.63^d^	49.78^cd^	52.00^b^	48.77^d^	50.65^bc^	54.23^a^	0.60	50.14	51.21	0.43	0.08	<0.001	0.11

^abcd^LSMeans ± SEM within a row of interaction means with different superscripts differ; *P* ≤ 0.05.

^xy^LSMeans ± SEM within a row of the main effect means with different superscripts differ; *P* ≤ 0.05.

^*^Improvest is a gonadotropin-releasing factor (GnRF) analog-diphtheria toxoid conjugate product approved for temporary suppression of ovarian function and estrus in market gilts (Zoetis Inc.); Improvest (IMP) gilts received the first Improvest injection on day 7 of the study (approximately 11 wk of age) and the second Improvest injection on day 67 of the study (approximately 19.5 wk of age); Control (CON) gilts did not receive Improvest injections; An equal number of CON and IMP gilts were marketed on day 89 (approximately 30% of the population), day 103 (approximately 30% of the population), and day 117 (approximately 40% of the population) of the study.

^†^Institutional meat purchase specifications (IMPS, 2014).

^‡^Sum of untrimmed ham and trimmed primal weights per carcass = [2 × (401A ham weight + 405A picnic weight + 406A butt weight + 409 belly weight + 414 loin weight + 416A spareribs weight)].

SEM, standard error of the mean.

Trimmed primal yields expressed as a percentage of HCW were presented in **Table 7**. There was a significant interaction (*P* ≤ 0.05) between Improvest management and the marketing group for the trimmed picnic when expressed as a percentage of HCW while there was not a significant interaction (*P* > 0.05) for the trimmed butt, trimmed, and squared belly, Canadian back loin, spareribs, or the sum of the trimmed primal cuts when expressed as a percentage of HCW. The Canadian back loin expressed as a percentage of HCW was 0.15 percentage units greater (*P* = 0.01) for CON gilts compared with IMP gilts. Other than this difference, there were no other effects (*P* > 0.05) for Improvest management on the trimmed primal cuts when expressed as a percentage of HCW. There was a significant difference (*P* < 0.01) among the marketing groups for all trimmed primal cut weights when expressed as a percentage of HCW with the 2 exceptions being for the Canadian back loin and, surprisingly, the sum of the trimmed primal cuts when expressed as a percentage of HCW.

**Table 7.** Effect of immunological suppression of ovarian function and estrus (Improvest) of market gilts marketed over 3 marketing groups (days 89 [22 d post-second dose], day 103 [37 d post-second dose], and day 117 of the study [day 50 post-second dose]) on trimmed (i.e., finished good/merchandized) primal weights expressed as a percentage of hot carcass weight (HCW)

	Interaction of Improvest Management and Marketing Group^*^							The main effect of improvest management					
	CON			IMP							*P*-value		
	day 89	day 103	day 117	day 89	day 103	day 117	SEM	CON	IMP	SEM	Improvest management	Marketing group	Interaction
Number of carcasses, no.	47	49	50	49	49	47		146	145				
Cut (IMPS #)^†^													
Picnic, trimmed (405A), % of HCW	8.18^c^	8.40^c^	9.19^a^	8.66^b^	8.38^c^	8.98^a^	0.10	8.59	8.67	0.05	0.28	<0.001	0.001
Butt, trimmed (406A), % of HCW	7.47^b^	8.18^a^	8.04^a^	7.65^b^	8.12^a^	8.17^a^	0.11	7.90	7.98	0.06	0.34	<0.001	0.51
Belly, trimmed and squared (409), % of HCW	12.36^a^	12.07^ab^	11.53^c^	12.22^a^	12.57^a^	11.59^bc^	0.19	11.99	12.13	0.12	0.40	<0.001	0.17
Loin, Canadian back (414), % of HCW	7.83^a^	7.72^ab^	7.64^ab^	7.66^ab^	7.54^b^	7.55^b^	0.07	7.73^x^	7.58^y^	0.04	0.016	0.09	0.83
Spareribs, trimmed (416A), % of HCW	5.05^ab^	4.91^bc^	5.12^a^	5.01^ab^	4.74^c^	5.15^a^	0.06	5.02	4.97	0.03	0.24	<0.001	0.26
Merchandized-cut yield^‡^, % of HCW	64.29^ab^	64.45^ab^	64.22^ab^	64.62^ab^	64.64^a^	63.97^b^	0.24	64.32	64.41	0.14	0.65	0.14	0.44

^abcd^LSMeans ± SEM within a row of interaction means with different superscripts differ; *P* ≤ 0.05.

^xy^LSMeans ± SEM within a row of the main effect means with different superscripts differ; *P* ≤ 0.05.

^*^Improvest is a gonadotropin-releasing factor (GnRF) analog-diphtheria toxoid conjugate product approved for temporary suppression of ovarian function and estrus in market gilts (Zoetis Inc.); Improvest (IMP) gilts received the first Improvest injection on day 7 of the study (approximately 11 wk of age) and the second Improvest injection on day 67 of the study (approximately 19.5 wk of age); Control (CON) gilts did not receive Improvest injections; An equal number of CON and IMP gilts were marketed on day 89 (approximately 30% of the population), day 103 (approximately 30% of the population), and day 117 (approximately 40% of the population) of the study.

^†^Institutional meat purchase specifications (IMPS, 2014).

^‡^Merchandized-cut yield = [(2 × (401A ham weight + 405A picnic weight + 406A butt weight + 408 belly weight + 414 loin weight + 416A spareribs weight)) / hot carcass weight] × 100.

SEM, standard error of the mean.

Trimmed primal yields expressed as a percentage of untrimmed primal weights were presented in **Table 8**. There was a significant interaction (*P* ≤ 0.05) between Improvest management and marketing group for the trimmed picnic as a percentage of the untrimmed picnic and for the trimmed spareribs as a percentage of the untrimmed spareribs. There were no effects (*P* > 0.05) for Improvest management on the untrimmed primal cuts when expressed as a percentage of the trimmed primal cuts with the lone exception of the spareribs which was 0.87 percentage units greater for CON gilts compared with IMP gilts. There was a significant difference (*P* < 0.01) among the marketing groups for all untrimmed primal cut weights when expressed as a percentage of trimmed primal cut weights with the lone exception for the Canadian back loin expressed as a percentage of the untrimmed loin.

**Table 8.** Effect of immunological suppression of ovarian function and estrus (Improvest) of market gilts marketed over 3 marketing groups (day 89 [22 d post-second dose], day 103 [37 d post-second dose], and day 117 of the study [day 50 post-second dose]) on yields of trimmed (i.e., finished good) primal weights calculated from untrimmed (i.e., natural fall) primal weights

	Interaction of Improvest Management and Marketing Group^1^							The main effect of improvest management					
	CON			IMP							*P*-value		
	day 89	day 103	day 117	day 89	day 103	day 117	SEM	CON	IMP	SEM	Improvest management	Marketing group	Interaction
Number of carcasses, no.	47	49	50	49	49	47		146	145				
Cut (IMPS #)^†^													
Picnic (405A), % of picnic (405)	80.83^c^	82.15^b^	82.29^ab^	81.25^c^	83.07^a^	81.62^bc^	0.33	81.76	81.98	0.19	0.41	<0.001	0.042
Butt (406A), % of butt (406)	86.48^b^	86.66^b^	87.61^a^	86.34^b^	86.61^b^	86.92^b^	0.25	86.92	86.62	0.14	0.15	0.001	0.35
Trimmed and squared belly (409), % of boneless belly (408)	66.24^a^	63.42^b^	63.55^b^	66.26^a^	64.99^ab^	63.57^b^	0.77	64.40	64.94	0.49	0.44	0.001	0.46
Canadian back loin (414), % of loin (410)	30.66	30.52	30.47	30.51	30.52	29.77	0.34	30.55	30.04	0.19	0.07	0.32	0.66
Trimmed spareribs (416A), % of spareribs (416)	88.51^b^	89.41^ab^	88.71^b^	90.06^a^	88.39^b^	85.58^c^	0.49	88.88^x^	88.01^y^	0.28	0.030	<0.001	<0.001

^abcd^LSMeans ± SEM within a row of interaction means with different superscripts differ; *P* ≤ 0.05.

^xy^LSMeans ± SEM within a row of the main effect means with different superscripts differ; *P* ≤ 0.05.

^*^Improvest is a gonadotropin-releasing factor (GnRF) analog-diphtheria toxoid conjugate product approved for temporary suppression of ovarian function and estrus in market gilts (Zoetis Inc.); Improvest (IMP) gilts received the first Improvest injection on day 7 of the study (approximately 11 wk of age) and the second Improvest injection on day 67 of the study (approximately 19.5 wk of age); Control (CON) gilts did not receive Improvest injections; An equal number of CON and IMP gilts were marketed on day 89 (approximately 30% of the population), day 103 (approximately 30% of the population), and day 117 (approximately 40% of the population) of the study.

^†^Institutional meat purchase specifications (IMPS, 2014).

SEM, standard error of the mean^.^

In total, these data suggest cutting yields between IMP and CON gilts (when the heaviest from each respective population are selected) were relatively similar and could be treated as such by the packing industry. Specifically, the primal-cut yields, merchandized cut yields, and trimmed cuts expressed as a percentage of untrimmed cuts between IMP and CON gilts were not statistically different from one another, and numerically were within 0.13% units, 0.10% units, and within 0.90% units of one another, respectively. It should be recognized that the lack of differences was observed in spite of greater backfat thickness for the IMP gilts compared with the CON gilts (1.8 mm fatter for the population in this study). The present study is the first study providing detailed cutting yield data between gilts managed with and without immunological suppression of ovarian function and estrus; therefore, limited data exist to make direct comparisons. It has been consistently reported that gilts managed with immunological suppression of ovarian function and estrus have greater backfat thickness when compared with untreated gilts (Bohrer et al., 2014; Poulsen-Nautrup et al., 2020; Pérez-Ciria et al., 2021). On a different note, Bohrer et al. (2023) reported that merchandized-cut yield was 1.52% units lower for physically castrated barrows when compared with untreated gilts. Thus, it can be assumed that gilts managed with immunological suppression of ovarian function and estrus should still hold carcass-cutting yield advantages over physically castrated barrows. This should be encouraging for the packing industry, particularly when producers adopt immunological suppression of GnRF for both male and female pigs (Improvest males have been reported to have a 1.41% unit advantage in lean cutting yields compared with physically castrated barrows; Harsh et al., 2017).

### Meat Quality

Meat quality attributes collected the day following slaughter (approximately 32-h postmortem) were presented in **Table 9**. There were no significant interactions (*P* ≥ 0.36) between Improvest management and the marketing group for pH or instrumental color in *longissimus* samples at 32-h postmortem. pH, instrumental L*, and instrumental b* were not affected by Improvest management, while instrumental a* was 0.32 units greater (*P *= 0.01) for CON gilts compared with IMP gilts (which is not likely a biologically meaningful magnitude; Zhu and Brewer, 1999). There were significant differences attributed to the marketing group for pH, instrumental a*, and instrumental b*; which can be summarized as marketing group #1 having a lower pH value, lower instrumental a* values, and instrumental b* values compared with marketing group #2 and marketing group # 3. The differences in meat quality parameters among marketing groups in this study were likely attributed to environmental conditions affected by different slaughter dates rather than the marketing group itself and should therefore be interpreted with caution. Arkfeld et al. (2017) reported marketing group contributed only 1.8% to the total variation in loin pH and did not contribute at all to the total variation for instrumental color of loins.

**Table 9.** Effect of immunological suppression of ovarian function and estrus (Improvest) of market gilts marketed over 3 marketing groups (day 89 [22 d post-second dose], day 103 [37 d post-second dose], and day 117 of the study [day 50 post-second dose]) on loin pH and instrumental color readings at 32-h postmortem

	Interaction of Improvest Management and Marketing Group^*^							The main effect of improvest management					
	CON			IMP							*P*-value		
	day 89	day 103	day 117	day 89	day 103	day 117	SEM	CON	IMP	SEM	Improvest management	Marketing group	Interaction
Number of loins, no.	47	49	50	49	49	47		146	145				
pH	5.53	5.64	5.63	5.51	5.64	5.64	0.02	5.60	5.59	0.01	0.82	<0.001	0.36
Minolta L* (lightness)	49.55	50.57	50.56	49.74	49.91	50.24	0.52	50.24	49.96	0.31	0.52	0.19	0.62
Minolta a* (redness)	5.44	5.68	5.93	5.13	5.47	5.48	0.15	5.68^x^	5.36^y^	0.09	0.014	0.014	0.68
Minolta b* (yellowness)	4.48	5.06	5.14	4.33	4.74	4.90	0.14	4.89	4.66	0.09	0.07	<0.001	0.82

^abcd^LSMeans ± SEM within a row of interaction means with different superscripts differ; *P* ≤ 0.05.

^xy^LSMeans ± SEM within a row of the main effect means with different superscripts differ; *P* ≤ 0.05.

^*^Improvest is a gonadotropin-releasing factor (GnRF) analog-diphtheria toxoid conjugate product approved for temporary suppression of ovarian function and estrus in market gilts (Zoetis Inc.); Improvest (IMP) gilts received the first Improvest injection on day 7 of the study (approximately 11 wk of age) and the second Improvest injection on day 67 of the study (approximately 19.5 wk of age); Control (CON) gilts did not receive Improvest injections; An equal number of CON and IMP gilts were marketed on day 89 (approximately 30% of the population), day 103 (approximately 30% of the population), and day 117 (approximately 40% of the population) of the study.

SEM, standard error of the mean^.^

Meat quality attributes collected following 14 d of postmortem aging were presented in **Table 10**. There were no significant interactions (*P* ≥ 0.06) between Improvest management and the marketing group for meat quality of *longissimus* samples at 14-d postmortem with the lone exception of IMF percentage. The interaction for IMF can also be described as differing magnitudes of change between CON and IMP gilts at each of the marketing groups. The IMP gilts marketed on day 89 had 0.2% units lower IMF compared to the CON gilts marketed on day 89, the IMP gilts marketed on day 103 had 0.61% units greater IMF compared to the CON gilts marketed on day 103, and the IMP gilts marketed on days 117 had 0.20% units greater IMF compared to the CON gilts marketed on day 117. There were no significant effects (*P* ≥ 0.12) for Improvest management of meat quality of *longissimus* samples at 14-d postmortem. The lack of differences in lean quality (i.e., purge loss, pH, instrumental color, and tenderness) between IMP and CON gilts was expected based on previous literature; however, it was surprising that there were no differences in IMF between IMP and CON gilts as gilts managed with immunological suppression of ovarian function and estrus usually have an improvement in IMF percentage (Pérez-Ciria et al., 2021; Bohrer et al., 2024; Vasquez-Hidalgo et al., 2024). There were no significant effects (*P* ≥ 0.06) for the main effect of the marketing group for meat quality of *longissimus* samples at 14-d postmortem with the 2 exceptions being pH and instrumental a*, which again were lower in their values for pigs in marketing group #1 compared with for pigs in marketing group #2 and marketing group #3.

**Table 10.** Effect of immunological suppression of ovarian function and estrus (Improvest) of market gilts marketed over 3 marketing groups (day 89 [22 d post-second dose], day 103 [37 d post-second dose], and day 117 of the study [day 50 post-second dose]) on purge loss, loin pH, instrumental color readings, intramuscular fat (IMF) composition, cooking loss, and star-probe following 14 d of postmortem storage

	Interaction of Improvest Management and Marketing Group^*^							The main effect of improvest management					
	CON			IMP							*P*-value		
	day 89	day 103	day 117	day 89	day 103	day 117	SEM	CON	IMP	SEM	Improvest management	Marketing group	Interaction
Number of loins, no.	20	20	19	20	19	18		59	57				
Purge loss, %	0.73	1.14	1.17	0.89	0.87	0.60	0.24	1.00	0.78	0.15	0.18	0.62	0.16
pH	5.65^c^	5.72^b^	5.71^b^	5.62^c^	5.77^a^	5.74^ab^	0.02	5.69	5.71	0.01	0.15	<0.001	0.06
Minolta L* (lightness)	49.61	50.41	48.91	50.26	49.28	49.23	0.52	49.64	49.59	0.29	0.90	0.17	0.17
Minolta a* (redness)	14.16	14.26	14.70	14.33	14.35	14.80	0.14	14.37	14.49	0.08	0.32	0.001	0.94
Minolta b* (yellowness)	3.48	3.47	3.45	3.49	3.29	3.54	0.10	3.46	3.44	0.06	0.78	0.44	0.38
Intramuscular fat (IMF), %	2.02^b^	1.90^b^	1.81^b^	1.82^b^	2.51^a^	2.01^b^	0.18	1.91	2.10	0.10	0.12	0.10	0.033
Star-probe, kg	4.64	4.83	4.71	4.67	4.76	4.50	0.16	4.73	4.64	0.12	0.44	0.34	0.63

^abcd^LSMeans ± SEM within a row of interaction means with different superscripts differ; *P* ≤ 0.05.

^xy^LSMeans ± SEM within a row of the main effect means with different superscripts differ; *P* ≤ 0.05.

^*^Improvest is a gonadotropin-releasing factor (GnRF) analog-diphtheria toxoid conjugate product approved for temporary suppression of ovarian function and estrus in market gilts (Zoetis Inc.); Improvest (IMP) gilts received the first Improvest injection on day 7 of the study (approximately 11 wk of age) and the second Improvest injection on day 67 of the study (approximately 19.5 wk of age); Control (CON) gilts did not receive Improvest injections; An equal number of CON and IMP gilts were marketed on day 89 (approximately 30% of the population), day 103 (approximately 30% of the population), and day 117 (approximately 40% of the population) of the study.

SEM, standard error of the mean^.^

In total, these data suggest that meat quality was not compromised when gilts were managed with immunological suppression of ovarian function and estrus. There were subtle differences between marketing groups, yet most of these differences could also be considered typical when observing meat quality parameters among sub-populations of pigs marketed in multiple groups.
